# Factors influencing TCM syndrome types of acute cerebral infarction: A binomial logistic regression analysis

**DOI:** 10.1097/MD.0000000000036080

**Published:** 2023-11-17

**Authors:** Shuning Zhang, Ji Yang

**Affiliations:** a The First Affiliated Hospital of Anhui University of Chinese Medicine, Center for Xin’an Medicine and Modernization of Traditional Chinese Medicine of IHM, Anhui University of Chinese Medicine, Hefei, China; b The First Affiliated Hospital of Anhui University of Chinese Medicine, Hefei, China.

**Keywords:** acute cerebral infarction, logistic regression analysis, real world, significant influencing factors, TCM syndrome classification

## Abstract

**Background:**

Acute cerebral infarction, characterized by a rapid onset and high fatality rate, presents a significant global challenge in terms of timely and effective treatment. In recent years, research focusing on the combined approach of traditional Chinese medicine (TCM) and Western medicine has demonstrated promising results in improving therapeutic outcomes in patients with acute cerebral infarction.

**Diagnosis:**

This study adhered to the latest edition of *Internal Medicine of Traditional Chinese Medicine*, published by the China Press of Traditional Chinese Medicine, as a reference. It selects eight commonly encountered TCM syndrome differentiations for accurate diagnosis.

**Methods:**

This study included 151 patients admitted to the hospital between 2019 and 2022 with acute cerebral infarction. Data on various diagnostic indicators were meticulously collected and subjected to single-factor analysis.

**Results:**

Among the multiple factors analyzed, those exhibiting a significance level of *P* < 0.05 included blood pressure, uric acid, glucose level, triglyceride level, total cholesterol level, homocysteine level, duration of disease, and cerebral infarction site. Subsequently, a binary logistic regression analysis was performed to assess the impact of these factors on different TCM syndrome types.

**Conclusion:**

The findings of this study indicate that Wind Phlegm Obstruction syndrome, triglyceride levels, location of cerebral infarction, uric acid levels, and disease duration significantly influence the development and progression of acute cerebral infarction. Additionally, blood pressure and cerebral infarction site were found to have a statistically significant impact on the Wind Yang Disturbance syndrome. Uric acid level and blood pressure were also identified as statistically significant factors. Moreover, total cholesterol and homocysteine levels were found to significantly affect phlegm stasis-blocking collateral syndrome. The insights gained from this study will contribute to the advancement of integrated treatment approaches, combining traditional Chinese and Western medicine, for acute cerebral infarction. Furthermore, these findings can serve as a valuable reference for the general population in terms of preventive measures against this condition.

## 1. Introduction

Acute cerebral infarction, also known as ischemic stroke, is a common clinical disease characterized by high morbidity, disability, mortality rates, sudden onset, dizziness, and other symptoms. It poses a significant threat to human health, and is a major focus of clinical research. The pathogenesis of acute cerebral infarction is complex and typically involves some form of trigger that disrupts blood supply to local brain tissue, affects oxygen supply, and causes irreversible damage that leads to necrosis and loss of neurological function. According to current research, >75% of cerebrovascular diseases are caused by acute cerebral infarction, which imposes a heavy burden on society and families and endangers human health. In recent years, significant progress has been made in Traditional Chinese medicine (TCM) research on acute cerebral infarction,^[1]^ particularly with regard to syndrome classification, which provides an objective basis for diagnosis and treatment and offers opportunities for combining Traditional Chinese medicine with western medicine.^[2]^

Traditional Chinese Medicine holds that the basic pathogenesis of acute cerebral infarction involves an imbalance of the viscera, reversal of qi and blood flow, intermingling of phlegm and blood stasis, and blockage of brain collaterals.^[3]^ The 6 most common causes are qi deficiency, phlegm accumulation, blood stasis, wind disturbance, fire excessiveness, and heat deficiency. In the acute stage, wind disturbances, phlegm accumulation, and blood stasis are more common. Traditional Chinese medicine has a long history of treating stroke and divides it into different syndrome types based on syndrome differentiation. Syndrome differentiation is a unique method for understanding, diagnosing, and treating diseases in traditional Chinese medicine that summarizes the etiology, nature, situation, and location of a disease at a certain stage.

In Western medicine, the treatment of cerebral infarction primarily involves thrombolysis, anticoagulation, and blood pressure regulation to alleviate symptoms and prevent further deterioration. Traditional Chinese medicine approaches the treatment of acute cerebral infarction through syndrome differentiation and treatment, using methods such as TCM decoctions, acupuncture, and rehabilitation training. In recent years, the prevention and treatment of acute cerebral infarction using integrated TCM and Western medicine have received much attention and have shown significant efficacy. Modern medicine has advanced research on the relationship between TCM syndrome types and objective clinical indicators.^[4]^ According to current clinical research, there are significant differences in the relevant test and examination results of patients with different degrees of cerebral infarction.^[5–9]^ Owing to the low requirements for equipment conditions and economic factors, medical examination, and inspection indicators are easily collected in clinical work. This study analyzed the correlation between TCM syndrome types and factors in the real world such as age, sex, blood pressure, uric acid, glucose levels, triglyceride levels, total cholesterol levels, low-density lipoprotein cholesterol levels, homocysteine levels, disease duration, and infarction site in 151 patients with acute cerebral infarction. The relationship between these factors and acute cerebral infarction and their influence on syndrome types was analyzed to provide a basis for the prevention and treatment of cerebral infarction using integrated TCM and Western medicine.

## 2. Materials and Methods

### 2.1. Research object

In this study, 151 patients admitted to the Department of Encephalopathy of the Geriatric Center of the First Affiliated Hospital of Anhui University of Traditional Chinese Medicine between 2019 and 2022 for acute cerebral infarction were retrospectively analyzed. Among the research subjects, there were 97 males with an average age of 69.3 years, and 54 females with an average age of 73.2 years.

### 2.2. Diagnostic criteria

The diagnostic criteria of Western medicine conform to those established by the latest edition of the Chinese Guidelines for the Diagnosis and Treatment of Acute Ischemic Stroke.^[10]^ These criteria include: (a) onset often occurs in a resting state; (b) most onsets do not have obvious headache or vomiting; (c) onset is relatively slow and usually progresses gradually or in stages; (d) consciousness is generally clear or mildly disturbed within 1–2 days after onset; (e) symptoms and signs of the internal carotid artery or basilar artery system; and (f) brain magnetic resonance examination should be performed, with cerebrospinal fluid from lumbar puncture generally not containing blood.

TCM diagnosis and syndrome classification standards are based on the diagnostic criteria and syndrome classification of stroke in the latest edition of “Internal Medicine of Traditional Chinese Medicine” published by the China Press of Traditional Chinese Medicine.^[11,12]^

### 2.3. Inclusion criteria

Inclusion criteria for this research object were as follows: (a) met the diagnostic criteria of acute cerebral infarction in Western medicine; (b) met the diagnostic criteria of stroke in traditional Chinese medicine; (c) age 18–102 years old, gender is not limited; (d) brain computed tomography or magnetic resonance imaging examination results showing responsible lesions, normal mental state, and complete relevant medical records after admission.

### 2.4. Exclusion criteria

(a) Patients with family genetic history; (b) patients who cannot judge TCM syndrome type; (c) pregnant or breastfeeding women; (d) patients with mental illness or unable to communicate; (e) patients with acute cerebral infarction admitted to hospital for more than 15 days after onset; (f) patients with malignant tumors, diabetic ketoacidosis, diabetic hyperosmolar coma, hematopoietic system diseases, and coagulation disorders; (g) severe heart, liver, kidney disease, or stroke caused by brain trauma, brain tumor, brain parasitic disease, metabolic disorder, rheumatic heart disease, coronary heart disease, and other heart diseases or combined with atrial fibrillation, arteritis, or blood disease; (h) patients with hemorrhagic stroke; (i) patients with definite infectious diseases in the past 2 weeks.

### 2.5. Rejection criteria

(a) Patients who are automatically discharged during treatment or whose data are incomplete; (b) sudden illnesses that cannot continue treatment.^[13]^

### 2.6. TCM dialectical classification

The TCM dialectical classification of the 151 patients included in this study was conducted according to the guidelines outlined in the latest edition of “Internal Medicine of Traditional Chinese Medicine,”^[11]^ published by the China Press of Traditional Chinese Medicine. A panel of 3 senior chief physicians comprehensively assessed and made judgments about each patient based on their clinical symptoms and corresponding examination results. To ensure the accuracy of the results, consensus among the 3 physicians was required for the final TCM syndrome differentiation of each patient. In cases where there was disagreement among physicians, another experienced chief physician was consulted for further research and judgment. Based on the above method, the final summary is classified into 8 categories of TCM syndrome differentiation types. These include wind phlegm obstruction syndrome (wind-phlegm blocking pattern, wind phlegm into collaterals type, wind phlegm and blood stasos syndrome), Wind Yang disturbance syndrome (wind-fire disturbance syndrome, liver and kidney deficiency syndrome), blood stasis syndrome (Qi and Yin deficiency with blood stasis syndrome), phlegm-stasis blocking collateral syndrome (phlegm and blood stasis syndrome, phlegm and blood stasis syndrome, phlegm and blood stasis syndrome, blood stasis syndrome, blood stasis syndrome, blood stasis syndrome), phlegm obstruction syndrome (wind phlegm disturbance syndrome), Yin deficiency wind syndrome (Yin deficiency yang hyperactivity syndrome), phlegm qi stagnation syndrome, and phlegm heat fu empirical.

### 2.7. Influencing factors

In this study, various factors, including traditional Chinese medicine (TCM) syndrome type, sex, age, blood pressure, uric acid, glucose level, triglyceride level, total cholesterol level, homocysteine level, low-density lipoprotein cholesterol level,^[14]^ and duration of disease were individually collected from a cohort of 151 patients with cerebral infarction. Additionally, the location of the cerebral infarction site was recorded. Based on this information, a comprehensive analysis and discussion can be conducted to explore the relationships and potential implications of these factors in the context of cerebral infarction.

### 2.8. Equipment and inspection methods

All patients were required to assume the supine position when undergoing cranial magnetic resonance imaging examinations and underwent routine cranial plain scans, with a slice thickness of 10 mm, a slice interval of 8 mm, and a scanning time of 20 to 30 minutes. Perform dynamic reexamination for some patients whose results are unclear or who require further examination to confirm the diagnosis.

### 2.9. Statistical analysis method

**Figure 1.**
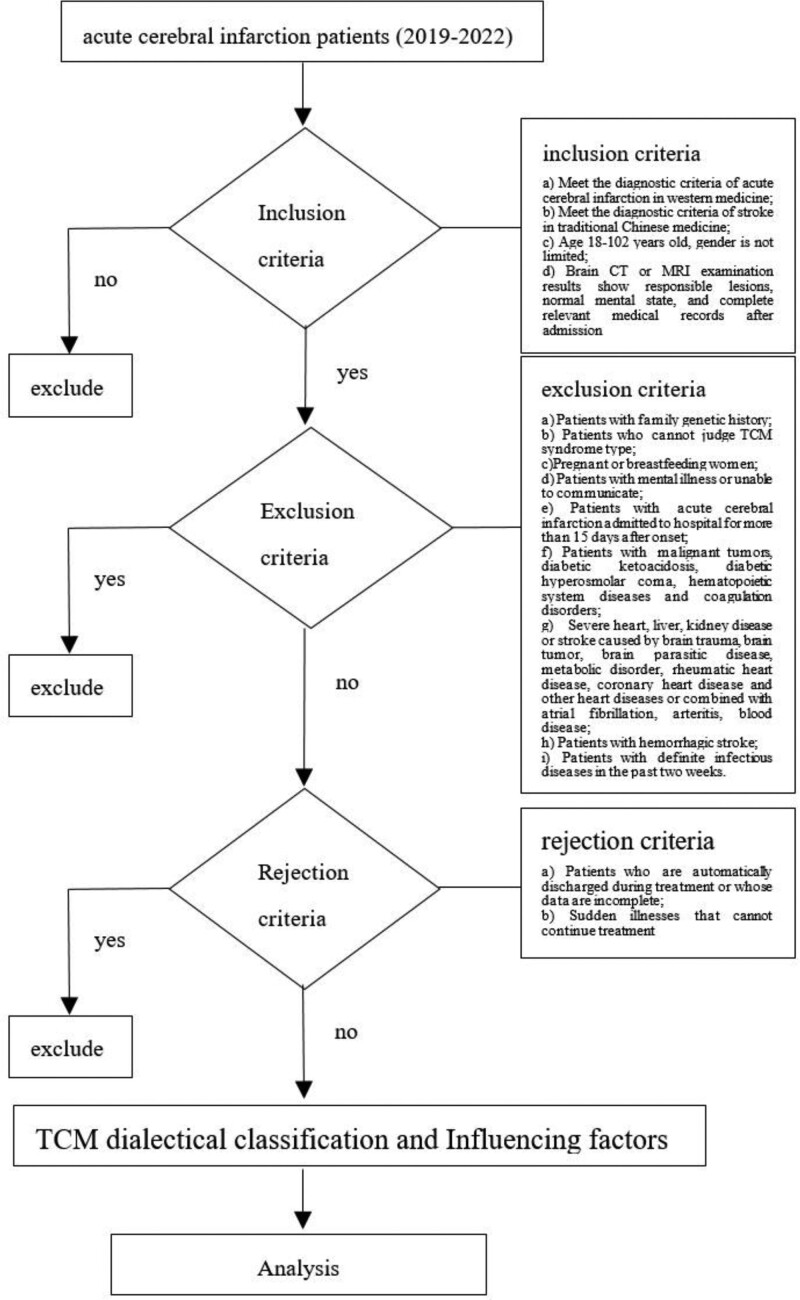
Study flow chart.

All data were statistically processed using the SPSS software (version 26.0). Enumeration data were expressed as case numbers and percentages, the chi-square test (*χ^2^*) was used, and Fisher exact test was used when the cell value was <5. *P* < .05, indicating that the difference between the 2 is statistically significant.^[15,16]^ First, according to the single factor analysis method, after identifying factors with statistical significance, binary logistic regression was used to explore the relationship between TCM syndrome types and different factors. The overall research process is shown in Figure 1.

## 3. Results

### 3.1. Basic statistics of inpatients

#### 3.1.1. Distribution of TCM syndrome types.

**Figure 2.**
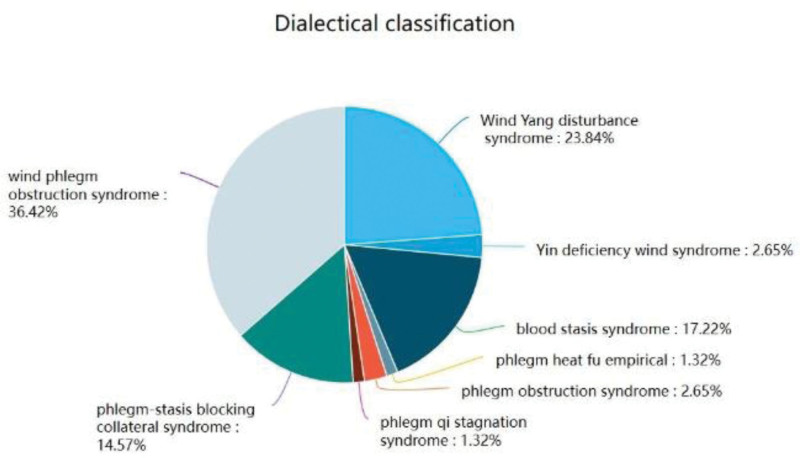
Dialectical classification.

The TCM syndrome types of 151 in patients with acute cerebral infarction were counted and the results are shown in Figure 2. Wind phlegm obstruction syndrome accounted for the highest proportion of 55 cases (36.42%), followed by Wind Yang disturbance syndrome in 36 cases (23.84%), blood stasis syndrome in 26 cases (17.22%), and phlegm stasis blocking collateral syndrome in 22 cases (14.57%), phlegm there were 4 cases of obstruction syndrome (2.65%), 4 cases of Yin deficiency wind syndrome (2.65%), 2 cases of phlegm qi stagnation syndrome (1.32%), and 2 cases of phlegm heat fu empirical (1.32%).

#### 3.1.2. Gender and age distribution.

**Figure 3.**
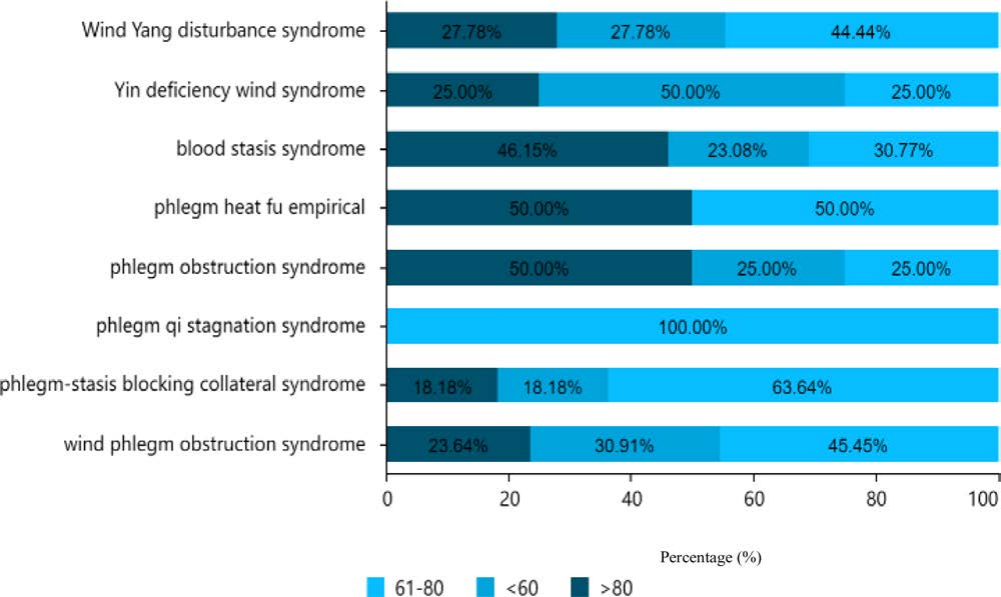
Distribution of traditional Chinese medicine syndrome types at different ages.

**Figure 4.**
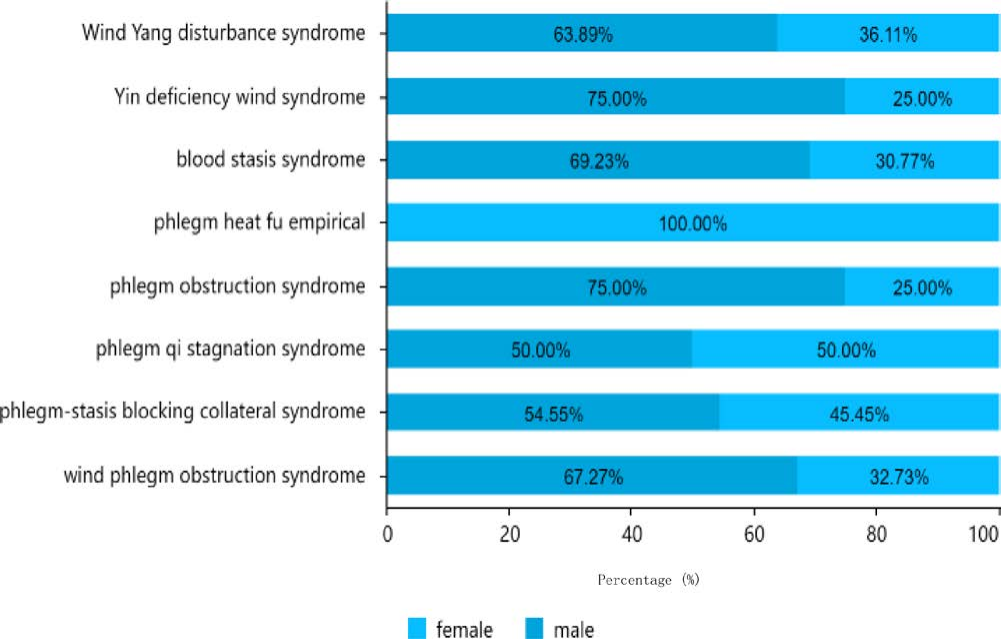
Distribution of traditional Chinese medicine syndrome types by gender.

As illustrated in Table 1, Figures 3, and 4, of the 151 cases analyzed, 97 (64.24%) were males and 54 (35.76%) were females, resulting in a male-to-female ratio of approximately 2:1. In terms of age distribution, most patients were aged between 61 to 80 years old, accounting for 68 cases (45.03%), followed by patients over the age of.

**Table 1:** Gender and age distribution.

		Wind Yang disturbance syndrome	Yin deficiency wind syndrome	Blood stasis syndrome	Phlegm heat fu empirical	Phlegm obstruction syndrome	Phlegm qi stagnation syndrome	Phlegm-stasis blocking collateral syndrome	Wind phlegm obstruction syndrome	Total
Gender	Female	13 (36.11%)	1 (25.00%)	8 (30.77%)	2 (100.00%)	1 (25.00%)	1 (50.00%)	10 (45.45%)	18 (32.73%)	54 (35.76%)
	Male	23 (63.89%)	3 (75.00%)	18 (69.23%)	0 (0.00%)	3 (75.00%)	1 (50.00%)	12 (54.55%)	37 (67.27%)	97 (64.24%)
Age	61–80	16 (44.44%)	1 (25.00%)	8 (30.77%)	1 (50.00%)	1 (25.00%)	2 (100.00%)	14 (63.64%)	25 (45.45%)	68 (45.03%)
	<60	10 (27.78%)	2 (50.00%)	6 (23.08%)	0 (0.00%)	1 (25.00%)	0 (0.00%)	4 (18.18%)	17 (30.91%)	40 (26.49%)
	>80	10 (27.78%)	1 (25.00%)	12 (46.15%)	1 (50.00%)	2 (50.00%)	0 (0.00%)	4 (18.18%)	13 (23.64%)	43 (28.48%)

Eighty patients had 43 cases (28.48%), and 40 patients were under the age of 60 years (26.49%). Among all age groups, the most common type was wind phlegm obstruction syndrome, with a total of 37 males and 18 females affected. Within this group, there were 25 patients aged between 61 to 80 years old, 17 patients were aged < 60 years, and 13 patients were aged > 80 years.

#### 3.1.3. Blood pressure distribution.

**Figure 5.**
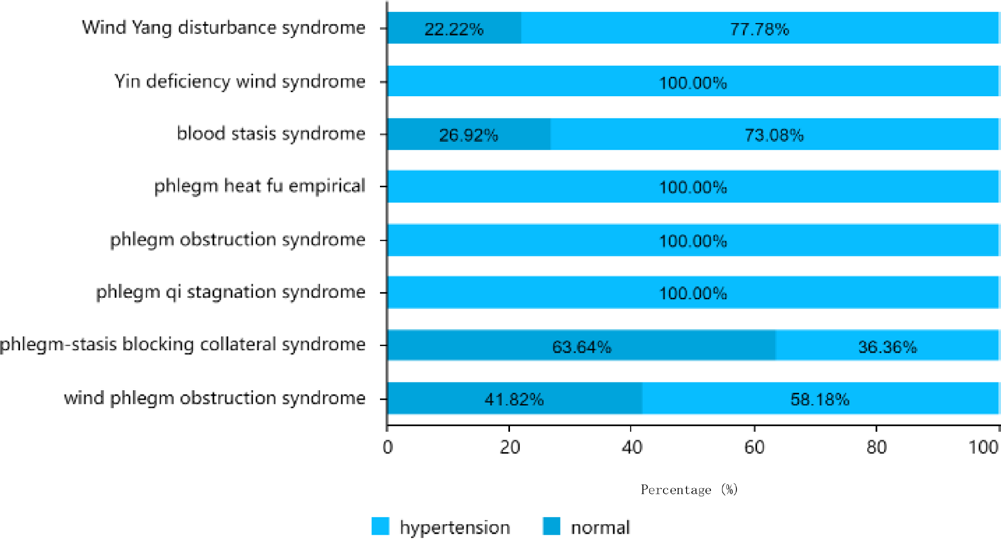
Traditional Chinese medicine syndrome distribution of different blood pressure.

As shown in Table 2, among the 151 cases, hypertensive patients accounted for the most, with a total of 99 cases (65.56%), among which the age of 61–80 was the highest, with a total of 46 cases; 31 cases were under 60 years old, and 80 years old and above. There are 22 cases. There were 52 patients (34.44%) had normal blood pressure, 22 of whom had aged 61 to 80, 21 patients were over 80 years old, and 9 patients were under 60 years old. There were 64 hypertensive and 33 normotensive cases among men, and 35 hypertensive and 19 normotensive women. The blood pressure distributions of the different TCM syndrome types are shown in Figure 5.

**Table 2:** Distribution of blood pressure and glucose distribution and triglyceride level distribution and total cholesterol level distribution and homocysteine level distribution and distribution of low-density lipoprotein cholesterol levels and by age and gender.

		High	Normal	Total
Blood pressure distribution				
Age	61–80	46 (46.46%)	22 (42.31%)	68 (45.03%)
	<60	31 (31.31%)	9 (17.31%)	40 (26.49%)
	>80	22 (22.22%)	21 (40.38%)	43 (28.48%)
Gender	female	35 (35.35%)	19 (36.54%)	54 (35.76%)
	male	64 (64.65%)	33 (63.46%)	97 (64.24%)
Glucose distribution				
Age	61–80	46 (46.46%)	22 (42.31%)	68 (45.03%)
	<60	31 (31.31%)	9 (17.31%)	40 (26.49%)
	>80	22 (22.22%)	21 (40.38%)	43 (28.48%)
Gender	female	35 (35.35%)	19 (36.54%)	54 (35.76%)
	male	64 (64.65%)	33 (63.46%)	97 (64.24%)
Triglyceride level distribution				
Age	61–80	19 (54.29%)	49 (42.24%)	68 (45.03%)
	<60	10 (28.57%)	30 (25.86%)	40 (26.49%)
	>80	6 (17.14%)	37 (31.90%)	43 (28.48%)
Gender	Female	14 (40.00%)	40 (34.48%)	54 (35.76%)
	Male	21 (60.00%)	76 (65.52%)	97 (64.24%)
Total cholesterol level distribution				
Age	61–80	17 (68.00%)	51 (40.48%)	68 (45.03%)
	<60	4 (16.00%)	36 (28.57%)	40 (26.49%)
	>80	4 (16.00%)	39 (30.95%)	43 (28.48%)
Gender	Female	10 (40.00%)	44 (34.92%)	54 (35.76%)
	Male	15 (60.00%)	82 (65.08%)	97 (64.24%)
Homocysteine level distribution				
Age	61–80	17 (43.59%)	51 (45.54%)	68 (45.03%)
	<60	10 (25.64%)	30 (26.79%)	40 (26.49%)
	>80	12 (30.77%)	31 (27.68%)	43 (28.48%)
Gender	Female	8 (20.51%)	46 (41.07%)	54 (35.76%)
	Male	31 (79.49%)	66 (58.93%)	97 (64.24%)
Distribution of low-density lipoprotein cholesterol levels				
Age	61–80	23 (60.53%)	45 (39.82%)	68 (45.03%)
	<60	7 (18.42%)	33 (29.20%)	40 (26.49%)
	>80	8 (21.05%)	35 (30.97%)	43 (28.48%)
Gender	Female	15 (39.47%)	39 (34.51%)	54 (35.76%)
	Male	23 (60.53%)	74 (65.49%)	97 (64.24%)

#### 3.1.4. Uric acid distribution.

**Figure 6.**
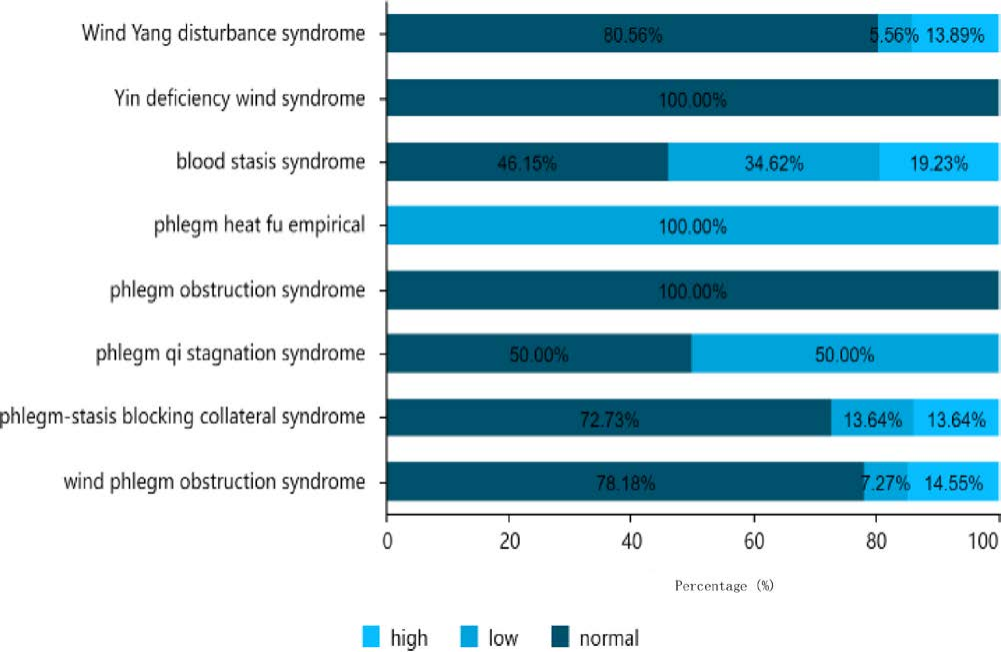
Traditional Chinese medicine syndrome distribution of uric acid.

As indicated in Table 3, of the 151 patients analyzed, the majority had normal uric acid levels, with a total of 109 cases (72.19%), including 71 males and 38 females. There were 21 patients with uric acid levels exceeding the standard value, comprising 18 males and 3 females. Additionally, 21 patients (8 men and 13 women) had uric acid levels lower than the standard value. Among patients aged between 61–80 years old, 50 had normal uric acid levels, 9 had high uric acid levels, and 9 had low uric acid levels. Among the patients aged <60 years, 30 had normal uric acid levels, 6 had high uric acid levels, and 4 had low uric acid levels. Among the patients aged >80 years, 29 had normal uric acid levels, 6 had high uric acid levels, and 8 had low uric acid levels. The distribution of uric acid in the different TCM syndrome types is shown in Figure 6.

**Table 3:** Distribution of uric acid by age and gender.

		High	Low	Normal	Total
Age	61–80	9 (42.86%)	9 (42.86%)	50 (45.87%)	68 (45.03%)
	<60	6 (28.57%)	4 (19.05%)	30 (27.52%)	40 (26.49%)
	>80	6 (28.57%)	8 (38.10%)	29 (26.61%)	43 (28.48%)
Gender	Female	3 (14.29%)	13 (61.90%)	38 (34.86%)	54 (35.76%)
	Male	18 (85.71%)	8 (38.10%)	71 (65.14%)	97 (64.24%)

#### 3.1.5. Glucose distribution.

**Figure 7.**
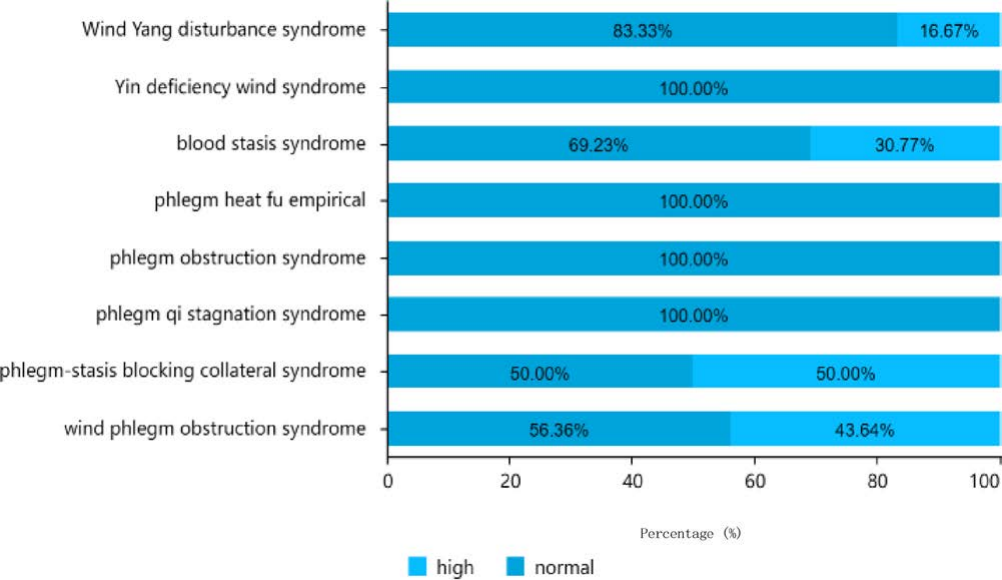
Traditional Chinese medicine syndrome distribution of glucose.

As indicated in Table 2, of the 151 patients analyzed, the majority had normal glucose levels, with a total of 102 cases (67.55%). This included 41 patients aged between 61–80 years old, 28 aged < 60 years, and 33 aged > 80 years. There were 49 patients (32.45%) had high glucose levels, comprising 27 patients aged—61 to 80 years old, 12 patients aged < 60 years, and 10 patients aged > 80 years. Among the male patients, 30 had high glucose levels and 67 had normal glucose levels, whereas among the female patients, 19 had high glucose levels and 35 had normal glucose levels. The distribution of glucose levels in the different TCM syndrome types is shown in Figure 7.

#### 3.1.6. Triglyceride level distribution.

**Figure 8.**
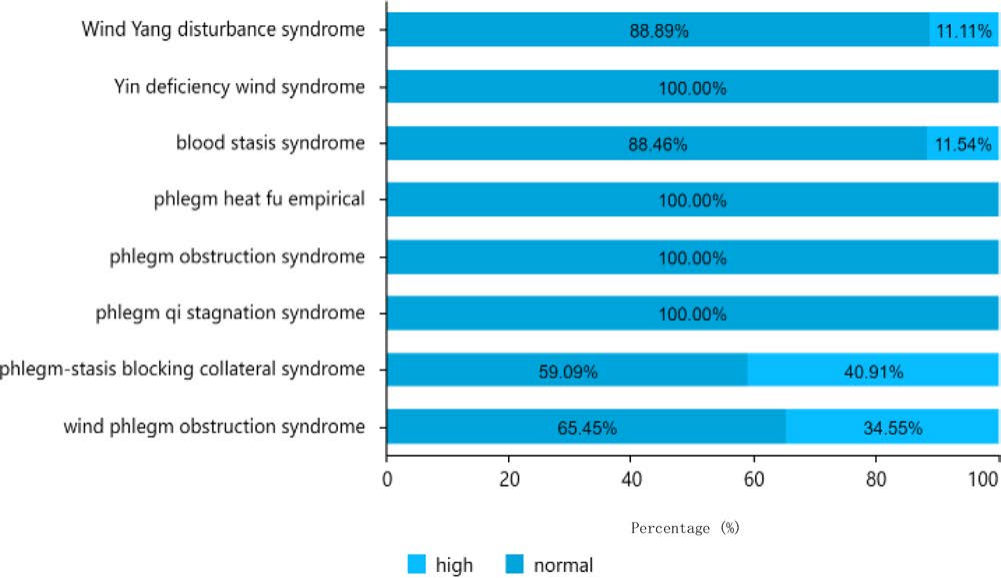
Traditional Chinese medicine syndrome distribution of triglyceride.

As indicated in Table 2, out of the 151 patients analyzed, the majority had normal triglyceride levels, with a total of 116 cases (76.82%), including 76 males and 40 females. There were 35 patients (23.18%) had high triglyceride levels, comprising 21 males and 14 females. Among patients aged between 61 to 80 years old, 19 had high triglyceride levels and 49 had normal levels. Among patients aged < 60 years, 10 had high triglyceride levels and 30 had normal levels. Among patients aged > 80 years, 6 had high triglyceride levels and 37 had normal levels. The distribution of triglyceride levels in different TCM syndrome types is shown in Figure 8.^[8]^

#### 3.1.7. Total cholesterol level distribution.

**Figure 9.**
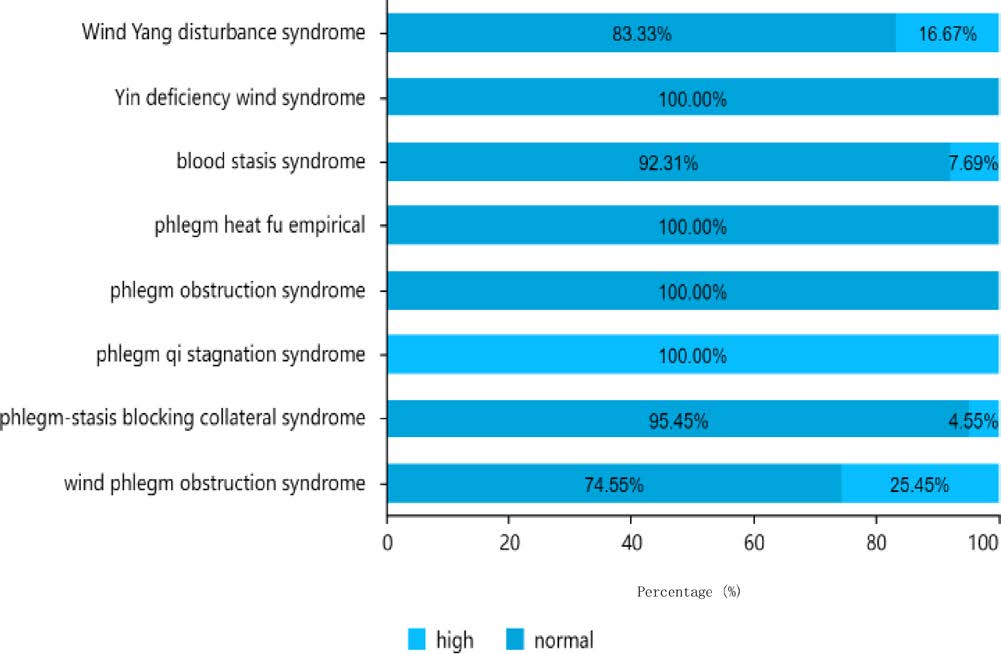
Traditional Chinese medicine syndrome distribution of total cholesterol.

As indicated in Table 2, of the 151 patients analyzed, the majority had normal total cholesterol levels, with a total of 126 cases (83.44%). This included 36 patients under the age of 60 years, 51 patients aged between 61 to 80 years old, and 39 patients aged > 80 years. There were 25 patients (16.56%) with high total cholesterol levels, comprising 4 patients under the age of 60, 17 patients aged between 61 to 80 years old, and 4 patients aged over 80 years. Among the male patients, 15 had high total cholesterol levels and 82 had normal levels, whereas among the female patients, 10 had high total cholesterol levels and 44 had normal levels. The distribution of total cholesterol levels in different TCM syndrome types is shown in Figure 9.

#### 3.1.8. Homocysteine level distribution.

**Figure 10.**
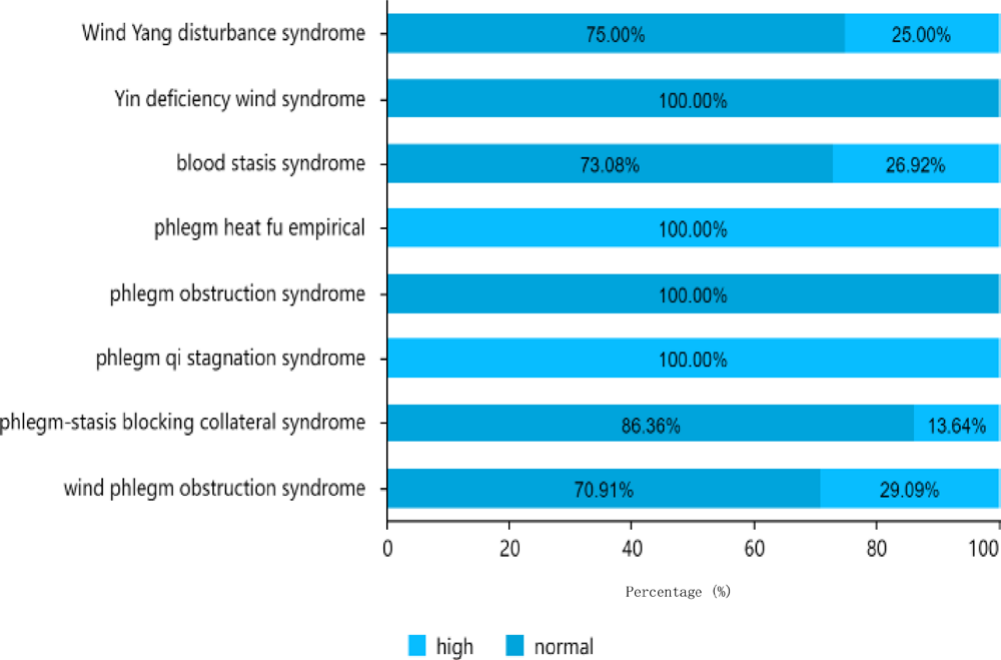
Traditional Chinese medicine syndrome distribution of homocysteine.

As indicated in Table 2, out of the 151 patients analyzed, the majority had normal homocysteine levels, with a total of 112 cases (74.17%), including 66 males and 46 females. There were 39 patients (25.83%) had high homocysteine levels, comprising 31 males and 8 females. Among patients aged between 61 to 80 years old, 17 had high homocysteine levels and 51 had normal levels. Among patients aged < 60 years, 10 had high homocysteine levels and 30 had normal levels. Among the patients aged > 80 years, 12 had high homocysteine levels and 31 had normal levels. The distribution of homocysteine levels in the different TCM syndrome types is shown in Figure 10.

#### 3.1.9. Time distribution of disease course.

**Figure 11.**
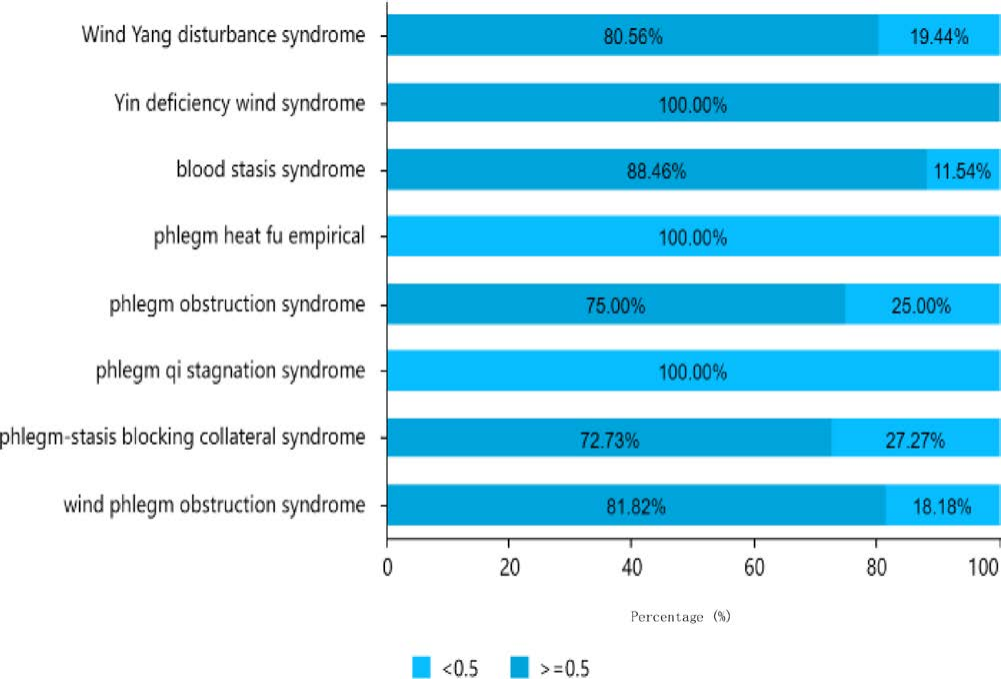
Traditional Chinese medicine syndrome distribution of disease duration.

As indicated in Table 4, out of the 151 patients analyzed, the majority had a disease duration of greater than or equal to 0.5 days, with a total of 120 cases (79.47%). This included 35 patients under the age of 60 years, 53 patients aged between 61 to 80 years old, and 32 patients aged over 80 years. There were 31 patients (20.53%) with a disease duration of <0.5 days, comprising 5 patients under the age of 60, 15 patients aged between 61 to 80 years old, and 11 patients aged over 80 years. Among the male patients, there were 17 cases with a disease duration of <0.5 days and 80 cases with a duration of greater than or equal to 0.5 days, while among the female patients, there were 14 cases with a duration of <0.5 days and 40 cases with a duration of greater than or equal to 0.5 days. The distribution of disease duration in different TCM syndrome types is shown in Figure 11.

**Table 4:** Distribution of disease duration by age and gender.

		<0.5	≥0.5	
Age	61–80	15 (48.39%)	53 (44.17%)	68 (45.03%)
	<60	5 (16.13%)	35 (29.17%)	40 (26.49%)
	>80	11 (35.48%)	32 (26.67%)	43 (28.48%)
Gender	Female	14 (45.16%)	40 (33.33%)	54 (35.76%)
	Male	17 (54.84%)	80 (66.67%)	97 (64.24%)

#### 3.1.10. Distribution of low-density lipoprotein cholesterol levels.

**Figure 12.**
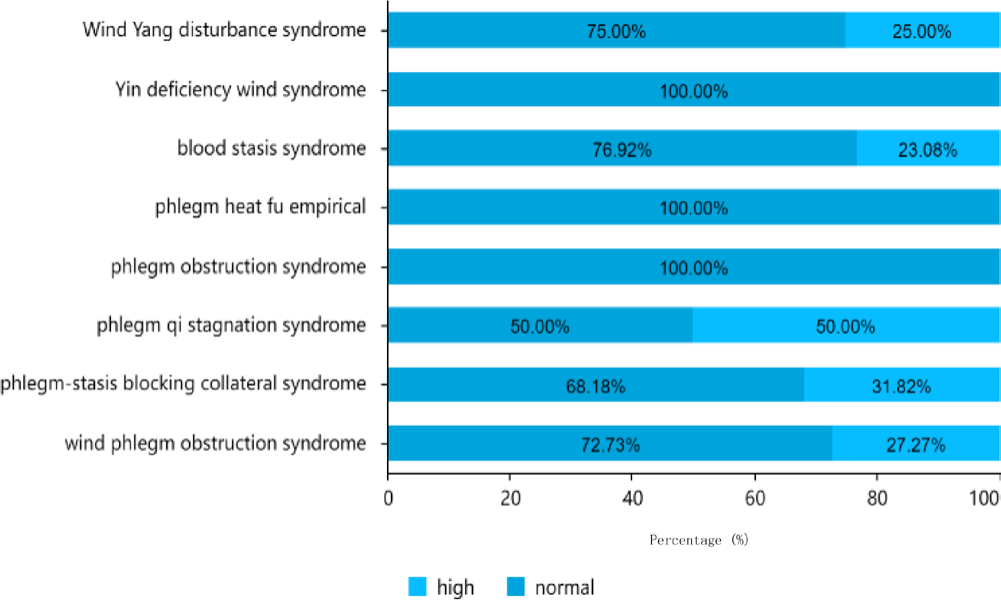
Traditional Chinese medicine syndrome distribution of low-density lipoprotein cholesterol.

As shown in Table 2, among the 151 patients, patients with normal low-density lipoprotein cholesterol levels were the most, with a total of 113 cases (74.83%), including 33 cases of patients under the age of 60, 45 cases of patients aged 61 to 80, and 45 cases of patients over 80 years old a total of 35 patients. There were 38 patients (25.17%) with high total cholesterol levels, including 7 patients under the age of 60, 23 patients aged 61 to 80, and 8 patients aged > 80 years. Among men, 23 had high low density lipoprotein (LDL) cholesterol levels and 74 had normal levels. There were 15 cases of women with high LDL cholesterol levels and 39 cases with normal LDL cholesterol levels. The distribution of low-density lipoprotein cholesterol levels in different TCM syndrome types is shown in Figure 12.

#### 3.1.11. Distribution of cerebral infarction sites.

Among the 151 patients involved in this study, the infarction sites were classified into: cerebral lobes (frontal lobe, parietal lobe, temporal lobe, occipital lobe, insula), ventricles (lateral ventricles, ventricles), brain stem (midbrain, pons, medulla), basal ganglia, others (semioval center, cingulate gyrus, hippocampus, internal capsule), thalamus, cerebellum, corpus callosum.

**Figure 13.**
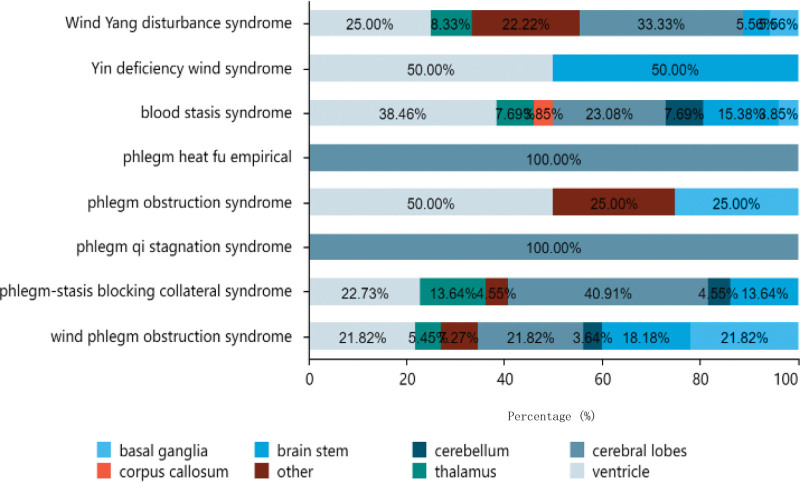
Traditional Chinese medicine syndrome distribution of cerebral infarction site.

As indicated in Table 5, out of the 151 patients analyzed, the majority had cerebral infarctions located in the lobe of the brain, with a total of 43 cases (28.48%), including 23 males and 20 females. This was followed by the ventricle, with a total of 40 cases (26.49%), including 28 males and 12 females; the brainstem, with 21 cases (13.91%), including 13 males and 8 females; the basal ganglia, with 16 cases (10.6%), including 13 males and 3 females; other parts, with 14 cases (9.27%), including 7 males and 7 females; the thalamus, with 11 cases (7.28%), including 8 males and 3 females; the cerebellum, with 5 cases (3.31%), including 4 males and 1 female; and the corpus callosum, with 1 case (0.66%), including 1 male and no females. Among patients aged between 61 to 80 years old, there were 7 infarcts in the basal ganglia, 15 in the brainstem, 2 in the cerebellum, 25 in the lobe of the brain, 5 in other parts, 3 in the thalamus, and 11 in the ventricles. Among patients aged < 60 years, there were 5 infarcts in the basal ganglia, 4 in the brainstem, none in the cerebellum or corpus callosum, 5 in the lobe of the brain, 3 in other parts, 6 in the thalamus, and 17 in the ventricles. Among patients over the age of 80 years, there were 4 infarcts in the basal ganglia, 2 in the brainstem, 3 in the cerebellum, none in the corpus callosum,13 in the lobe of the brain,6 in other parts,2 in the thalamus, and 12 in the ventricle. Distribution of cerebral infarction sites among different TCM syndromes types are illustrated in Figure 13.

**Table 5:** Traditional Chinese medicine syndrome distribution of cerebral infarction site.

		Basal ganglia	Brain stem	Cerebellum	Cerebral lobes	Corpus callosum	Other	Thalamus	Ventricle	Total
Age	61–80	7 (43.75%)	15 (71.43%)	2 (40.00%)	25 (58.14%)	0 (0.00%)	5 (35.71%)	3 (27.27%)	11 (27.50%)	68 (45.03%)
	<60	5 (31.25%)	4 (19.05%)	0 (0.00%)	5 (11.63%)	0 (0.00%)	3 (21.43%)	6 (54.55%)	17 (42.50%)	40 (26.49%)
	>80	4 (25.00%)	2 (9.52%)	3 (60.00%)	13 (30.23%)	1 (100.00%)	6 (42.86%)	2 (18.18%)	12 (30.00%)	43 (28.48%)
Gender	Female	3 (18.75%)	8 (38.10%)	1 (20.00%)	20 (46.51%)	0 (0.00%)	7 (50.00%)	3 (27.27%)	12 (30.00%)	54 (35.76%)
	Male	13 (81.25%)	13 (61.90%)	4 (80.00%)	23 (53.49%)	1 (100.00%)	7 (50.00%)	8 (72.73%)	28 (70.00%)	97 (64.24%)

### 3.2. Univariate analysis of different TCM syndrome types and related factors

To explore these selected factors, they were found to have a significant correlation with TCM syndrome types. Next, this study conducted a univariate analysis of all factors to screen out statistically significant factors.

As indicated in Table 6, the results of *χ^2^* test showed that some of the factors had no statistically significant differences among the different TCM syndrome types. Including age (*χ^2^* = 12.671, *P* = .488 > .05), gender (*χ^2^* = 5.337, *P* = .641 > 0.05), and low-density lipoprotein cholesterol (*χ^2^* = 3.813, *P* = .82 > .05).

**Table 6:** Univariate analysis result.

		Wind Yang disturbance syndrome	Yin deficiency wind syndrome	Blood stasis syndrome	Phlegm heat fu empirical	Phlegm obstruction syndrome	Phlegm qi stagnation syndrome	Phlegm-stasis blocking collateral syndrome	Wind phlegm obstruction syndrome	Total	χ2	*P*
Age	61–80	16 (44.44)	1 (25.00)	8 (30.77)	1 (50.00)	1 (25.00)	2 (100.00)	14 (63.64)	25 (45.45)	68 (45.03)	12.671	.488
	<60	10 (27.78)	2 (50.00)	6 (23.08)	0 (0.00)	1 (25.00)	0 (0.00)	4 (18.18)	17 (30.91)	40 (26.49)		
	>80	10 (27.78)	1 (25.00)	12 (46.15)	1 (50.00)	2 (50.00)	0 (0.00)	4 (18.18)	13 (23.64)	43 (28.48)		
Gender	Female	13 (36.11)	1 (25.00)	8 (30.77)	2 (100.00)	1 (25.00)	1 (50.00)	10 (45.45)	18 (32.73)	54 (35.76)	5.337	.641
	Male	23 (63.89)	3 (75.00)	18 (69.23)	0 (0.00)	3 (75.00)	1 (50.00)	12 (54.55)	37 (67.27)	97 (64.24)		
Blood pressure	Hypertension	28 (77.78)	4 (100.00)	19 (73.08)	2 (100.00)	4 (100.00)	2 (100.00)	8 (36.36)	32 (58.18)	99 (65.56)	16.635	**.008**
	Normal	8 (22.22)	0 (0.00)	7 (26.92)	0 (0.00)	0 (0.00)	0 (0.00)	14 (63.64)	23 (41.82)	52 (34.44)		
Uric acid	High	5 (13.89)	0 (0.00)	5 (19.23)	0 (0.00)	0 (0.00)	0 (0.00)	3 (13.64)	8 (14.55)	21 (13.91)	28.928	**.015**
	Low	2 (5.56)	0 (0.00)	9 (34.62)	2 (100.00)	0 (0.00)	1 (50.00)	3 (13.64)	4 (7.27)	21 (13.91)		
	Normal	29 (80.56)	4 (100.00)	12 (46.15)	0 (0.00)	4 (100.00)	1 (50.00)	16 (72.73)	43 (78.18)	109 (72.19)		
Glucose	High	6 (16.67)	0 (0.00)	8 (30.77)	0 (0.00)	0 (0.00)	0 (0.00)	11 (50.00)	24 (43.64)	49 (32.45)	14.34	**.024**
	Normal	30 (83.33)	4 (100.00)	18 (69.23)	2 (100.00)	4 (100.00)	2 (100.00)	11 (50.00)	31 (56.36)	102 (67.55)		
Triglyceride	High	4 (11.11)	0 (0.00)	3 (11.54)	0 (0.00)	0 (0.00)	0 (0.00)	9 (40.91)	19 (34.55)	35 (23.18)	16.418	**.022**
	Normal	32 (88.89)	4 (100.00)	23 (88.46)	2 (100.00)	4 (100.00)	2 (100.00)	13 (59.09)	36 (65.45)	116 (76.82)		
Total cholesterol	High	6 (16.67)	0 (0.00)	2 (7.69)	0 (0.00)	0 (0.00)	2 (100.00)	1 (4.55)	14 (25.45)	25 (16.56)	13.64	**.033**
	Normal	30 (83.33)	4 (100.00)	24 (92.31)	2 (100.00)	4 (100.00)	0 (0.00)	21 (95.45)	41 (74.55)	126 (83.44)		
Homocysteine	High	9 (25.00)	0 (0.00)	7 (26.92)	2 (100.00)	0 (0.00)	2 (100.00)	3 (13.64)	16 (29.09)	39 (25.83)	16.315	**.022**
	Normal	27 (75.00)	4 (100.00)	19 (73.08)	0 (0.00)	4 (100.00)	0 (0.00)	19 (86.36)	39 (70.91)	112 (74.17)		
Course of disease days	<0.5	7 (19.44)	0 (0.00)	3 (11.54)	2 (100.00)	1 (25.00)	2 (100.00)	6 (27.27)	10 (18.18)	31 (20.53)	13.9	**.029**
	≥0.05	29 (80.56)	4 (100.00)	23 (88.46)	0 (0.00)	3 (75.00)	0 (0.00)	16 (72.73)	45 (81.82)	120 (79.47)		
Low-density Lipoprotein cholesterol	High	9 (25.00)	0 (0.00)	6 (23.08)	0 (0.00)	0 (0.00)	1 (50.00)	7 (31.82)	15 (27.27)	38 (25.17)	3.813	.82
	Normal	27 (75.00)	4 (100.00)	20 (76.92)	2 (100.00)	4 (100.00)	1 (50.00)	15 (68.18)	40 (72.73)	113 (74.83)		
Cerebral infarction site	Basal ganglia	2 (5.56)	0 (0.00)	1 (3.85)	0 (0.00)	1 (25.00)	0 (0.00)	0 (0.00)	12 (21.82)	16 (10.60)	62.577	**.045**
	Brain stem	2 (5.56)	2 (50.00)	4 (15.38)	0 (0.00)	0 (0.00)	0 (0.00)	3 (13.64)	10 (18.18)	21 (13.91)		
	Cerebellum	0 (0.00)	0 (0.00)	2 (7.69)	0 (0.00)	0 (0.00)	0 (0.00)	1 (4.55)	2 (3.64)	5 (3.31)		
	Cerebral lobes	12 (33.33)	0 (0.00)	6 (23.08)	2 (100.00)	0 (0.00)	2 (100.00)	9 (40.91)	12 (21.82)	43 (28.48)		
	Corpus callosum	0 (0.00)	0 (0.00)	1 (3.85)	0 (0.00)	0 (0.00)	0 (0.00)	0 (0.00)	0 (0.00)	1 (0.66)		
	Other	8 (22.22)	0 (0.00)	0 (0.00)	0 (0.00)	1 (25.00)	0 (0.00)	1 (4.55)	4 (7.27)	14 (9.27)		
	Thalamus	3 (8.33)	0 (0.00)	2 (7.69)	0 (0.00)	0 (0.00)	0 (0.00)	3 (13.64)	3 (5.45)	11 (7.28)		
	Ventricle	9 (25.00)	2 (50.00)	10 (38.46)	0 (0.00)	2 (50.00)	0 (0.00)	5 (22.73)	12 (21.82)	40 (26.49)		

The bold values in Table 6 indicate values less than 0.05, which are statistically significant.

Some of the factors showed statistically significant differences among the different TCM syndrome types. Including blood pressure (*χ^2^* = 16.635, *P* = .008 < .05), uric acid (*χ^2^* = 28.928, *P* = .015 < .05), glucose (*χ^2^* = 14.34, *P* = .024 < .05), triglyceride (*χ^2^* = 16.418, *P* = .022 < .05), total cholesterol (*χ^2^* = 14.34, *P* = .026 < .05), homocysteine (*χ^2^* = 16.315, *P* = .022 < .05), course of disease days (*χ^2^* = 13.9, *P* = .029 < .05), and cerebral infarction sites (*χ^2^* = 62.577, *P* = .045 < .05).

### 3.3. Logistic regression analysis of different TCM syndrome types and related factors

Univariate analysis revealed that among all the patient factors, blood pressure, uric acid, glucose, triglycerides, total cholesterol, homocysteine, disease duration, and infarction site had statistically significant differences in distribution among different syndrome types. Therefore, a binary logistic regression analysis was performed using different TCM syndrome types as dependent variables and the selecting factors (*P* < .05) in the univariate analysis as independent variables (blood pressure, uric acid, glucose, triglycerides, total cholesterol, homocysteine, disease duration, and infarction site). The distribution of syndrome types and general characteristics in Wind Yang disturbance syndrome and non-wind yang disturbance syndrome, Yin deficiency wind syndrome and non-Yin deficiency wind syndrome, blood stasis syndrome and non-blood stasis syndrome, phlegm heat fu empirical and non-phlegm heat fu empirical, phlegm obstruction syndrome and non-phlegm obstruction syndrome, phlegm qi stagnation syndrome and non-phlegm qi stagnation syndrome, phlegm stasis blocking collateral syndrome, and non-phlegm stasis blocking collateral syndrome, wind phlegm obstruction syndrome, and non-wind phlegm obstruction syndrome are discussed.

The results of the regression analysis are shown in Table 7.

**Table 7:** Summary of binary logistic regression analysis results.

	Regression coefficients	Wald χ^2^	*P*	OR	95%CI
Wind Yang disturbance syndrome and non-Wind Yang disturbance syndrome					
Blood Pressure	−2.49	3.938	.047	0.083	0.007–0.970
Cerebral infarction site	1.678	5.134	.023	5.355	1.254–22.865
Blood stasis syndrome and non-blood stasis syndrome					
Uric acid	−0.943	9.134	.003	0.39	0.211–0.718
Blood Pressure	−1.38	6.061	.014	0.252	0.084–0.755
Phlegm-stasis blocking collateral syndrome and non-phlegm-stasis blocking collateral syndrome					
Total Cholesterol	−0.413	4.93	.026	0.867	0.765–0.983
Homocysteine	1.436	7.136	.008	4.203	1.466–12.052
Wind phlegm obstruction syndrome and non-wind phlegm obstruction syndrome					
Triglyceride	−0.909	3.93	.047	0.403	0.164–0.990
Cerebral infarction site	−0.192	5.864	.015	0.826	0.707–0.964
Uric Acid	−1.345	3.955	.047	0.261	0.069–0.981
Course of disease days	−0.937	5.428	.02	0.392	0.178–0.862

In the regression equation constructed with Wind Yang disturbance syndrome and non-Wind Yang disturbance syndrome as dependent variables, blood pressure had a statistically significant effect on Wind Yang disturbance syndrome (OR = 0.083, 95%CI 0.007–0.970, *P* = .047), indicating that an increase in blood pressure increases the probability of Wind Yang disturbance syndrome. The infarction site also had a statistically significant effect on Wind Yang disturbance syndrome (OR = 5.355, 95%CI 1.254–22.865, *P* = .023), indicating that the infarction site would increase the probability of Wind Yang disturbance syndrome.

In the regression equation constructed with blood stasis syndrome and non-blood stasis syndrome as dependent variables, uric acid had a statistically significant effect on blood stasis syndrome (OR = 0.39, 95%CI 0.211–0.718, *P* = .003), indicating that an increase in uric acid levels would increase the probability of blood stasis syndrome. Blood pressure also had a statistically significant effect on blood stasis syndrome (OR = 0.252, 95%CI 0.084–0.755, *P* = .014), indicating that an increase in blood pressure increases the probability of blood stasis syndrome.

In the regression equation constructed with phlegm-stasis blocking collateral syndrome and non-phlegm-stasis blocking collateral syndrome as dependent variables, total cholesterol had a statistically significant effect on phlegm-stasis blocking collateral syndrome (OR = 0.867, 95%CI 0.765–0.983, *P* = .026), indicating that an increase in total cholesterol levels would increase the probability of phlegm-stasis blocking collateral syndrome. Homocysteine also had a statistically significant effect on phlegm-stasis blocking collateral syndrome (OR = 4.203, 95%CI 1.466–12.052, *P* = .008), indicating that an increase in homocysteine levels would increase the probability of phlegm-stasis blocking collateral syndrome.

In the regression equation constructed with wind phlegm obstruction syndrome and non-wind phlegm obstruction syndrome as dependent variables, triglycerides had a statistically significant effect on wind phlegm obstruction syndrome (OR = 0.403, 95%CI 0.164–0.990, *P* = .047), indicating that an increase in triglyceride levels would increase the probability of wind phlegm obstruction syndrome. The infarction site also had a statistically significant effect on wind phlegm obstruction syndrome (OR = 0.826, 95%CI 0.707–0.964, *P* = .015), indicating that the location of the infarction increased the probability of wind phlegm obstruction syndrome. Uric acid had a statistically significant effect on wind phlegm obstruction syndrome (OR = 0.261, 95%CI 0.069–0.981, *P* = .047), indicating that an increase in uric acid levels would increase the probability of wind phlegm obstruction syndrome. Disease duration also had a statistically significant effect on wind phlegm obstruction syndrome (OR = 0.392, 95%CI 0.178–0.862, *P* = .020), indicating that an increase in disease duration would increase the probability of wind phlegm obstruction syndrome.

## 4. Discuss

The distribution of TCM syndrome types in acute cerebral infarction follows a certain pattern. In descending order of distribution ratio, the syndrome types were wind phlegm obstruction syndrome, Wind Yang disturbance syndrome, blood stasis syndrome, phlegm stasis blocking collateral syndrome, Yin-deficiency Wind syndrome, phlegm obstruction syndrome, phlegm heat fu empirical, and phlegm qi stagnation syndrome.

Statistical analysis revealed that several factors were significantly associated with the 8 TCM syndrome types. These factors include blood pressure, uric acid level, glucose level, triglyceride level, total cholesterol level, homocysteine level, disease duration, and cerebral infarction site.

Wind phlegm obstruction syndrome is currently the most common syndrome in acute cerebral infarction, with most patients having a disease duration of greater than or equal to 0.5 days. According to previous research theories,^[9,17,18]^ some biochemical indicators are related to acute cerebral infarction. Real-world case studies have confirmed that factors such as triglyceride level, cerebral infarction site, uric acid level, and disease duration have statistical significance.

The distribution ratio of Wind Yang disturbance syndrome is second only to that of wind phlegm obstruction syndrome. Most patients with Wind Yang have normal levels of total cholesterol, triglycerides, glucose, and uric acid. Factors such as blood pressure and infarction site had significant effects on this syndrome type.

The age distribution of patients with blood stasis syndrome is relatively balanced, with similar numbers of patients under 60, 60 to 80 years old, and over 80 years of age. Factors such as uric acid level and blood pressure had statistically significant effects on this syndrome type.

The number of patients with phlegm stasis-blocking collateral syndrome was similar between males and females. Factors such as total cholesterol and homocysteine levels had statistically significant effects on this syndrome type.

For patients diagnosed with yin deficiency wind syndrome, phlegm obstruction syndrome, phlegm heat fu empirical, and phlegm qi stagnation syndrome, the overall probability was greater for males than for females. The age distribution of the patients was relatively balanced.

In summary, the distribution of TCM syndrome types in acute cerebral infarction is correlated with the physical and chemical indicators of patients, and can reflect the objective characteristics of TCM syndrome types to a certain extent. All case information included in this study came from inpatient cases in the Department of Encephalology at the Geriatrics Center of the Anhui Provincial Hospital of Traditional Chinese Medicine. Although the selection was as random and objective as possible, there were still issues of clinical data and selection bias to some extent. In future studies, we will expand the sample size and selection range as much as possible. In this study, experts strictly classified patients according to the latest publication of “Internal Medicine of Traditional Chinese Medicine.” Although the selected 8 TCM syndrome types cannot fully reflect the actual condition of patients, they cover most TCM syndrome types in acute cerebral infarction.

## Author contributions

**Data curation:** Shuning Zhang.

**Formal analysis:** Shuning Zhang.

**Investigation:** Shuning Zhang.

**Methodology:** Shuning Zhang.

**Project administration:** Shuning Zhang, Ji Yang.

**Resources:** Ji Yang.

**Software:** Ji Yang.

**Writing – original draft:** Shuning Zhang.

**Writing – review & editing:** Shuning Zhang.
